# Seizures, Psychosis, and Cerebral Vascular Malformation: A Rare Chain of Events

**DOI:** 10.1155/2024/8656715

**Published:** 2024-01-22

**Authors:** Ismail Hanine, Khadija Benallel, Roukaya Benjelloun, Mohamed Kadiri

**Affiliations:** ^1^Mohamed V Military Hospital of Rabat, Mohamed V University of Rabat, Rabat, Morocco; ^2^Mohammed VI University of Health Sciences of Casablanca, Casablanca, Morocco

## Abstract

**Background:**

In psychiatry, anatomical abnormalities are sometimes forgotten, and this can mislead doctors into thinking that the diagnosis is purely psychiatric. A physical examination is important whenever it is possible. Even though cerebral arteriovenous malformations (cAVMs) are rare and can go unnoticed, in some cases they can cause clinical symptoms, which is a complication. *Case Presentation*. In this case, we describe a patient with no prior medical or psychiatric history having a cAVM diagnosed after showing psychotic symptoms (delusion and disorganized thoughts and behavior). The deep 4 × 5 cm cAVM was discovered after admitting the patient to psychiatric ward, the neurological cause has been considered after a recorded seizure, which brings the following question: Is the clinical presentation a direct result of the cAVM or is it postictal?

**Conclusions:**

An abnormality leading to another, here is how we could describe our patient's psychopathology leading to psychotic symptoms. The two hypotheses explaining this case report have a low rate of occurring making this a rare case. Either way, neurological cause cannot be overlooked even if the clinical presentation is typical.

## 1. Introduction

Delusions, hallucinations, and disorganized thoughts are among the most frequent symptoms of a schizophrenic spectrum disorder, especially if the patient is young and does not have any prior history of a mental disorder and no other somatic symptoms. Nevertheless, an organic condition, although unlikely, should always be ruled out.

Although being named “*Psychotic disorders due to another medical condition*” in the DSM-5 [1], some researches still refer to them as “secondary psychoses” from the DSM-IV-TR [2] or “organic psychoses”.

There frequency is difficult to assess since there is a variety of causes, two studies estimated the prevalence of organic psychotic disorders to be between 0.21% and 0.57% [3, 4].

Those disorders should be suspected, based on specific clinical, radiological or laboratory investigations. The late onset of the psychosis, finding some dominant features such as confusion, seizures, catatonia or visual hallucinations, kidney failure, metabolic disorders, and B vitamins deficiency increase the likelihood of organic psychosis [5].

We aim to emphasize these diagnoses through the case of a 20-year-old patient, without any neurological or psychiatric history, who presented a disorganization syndrome with delusions of persecution. The evaluation of this purely psychiatric presentation revealed a left frontal cerebral arteriovenous malformation (cAVM) of 4 × 5 cm after a clonic seizure.

This case illustrates the need to systematically investigate any acute onset psychiatric presentation, even if it looks typical, in order to rule out an organic cause, which may be neurological, vascular, or something else.

We are focusing on these investigations since in most cases, on one hand the prevalence of cerebral vascular abnormalities in the general population is relatively rare (0.06% to 0.5%) are almost always asymptomatic, and frontal lobe are more associated with mood disorders. and on the other hand, in post-ictal psychosis overall, affective symptoms are more frequent then first rank (Schneiderian) symptoms of schizophrenia.

## 2. Case Presentation

M. is a 20-year-old single patient with no prior medical, surgical, or psychiatric history, no substance use, and no family health history besides a diabetic mother (no epilepsy, psychotic disorder nor cerebral vascular abnormalities). He was transferred to the psychiatric emergency room for a recent behavioral change.

One week prior to his admission, while traveling to his new workplace, which is 300 km from his home. The patient got off the bus he was traveling in, halfway through his trip and wandered aimlessly in an unfamiliar city. After several days of wandering and begging, the patient was able to reach his destination with the help of other Samaritans.

Upon arrival at his workplace, the patient was in a state of neglect, expressing delusional thoughts and behaving in a bizarre manner. He was therefore taken to the psychiatric emergency room for treatment.

During his transfer, the patient presented a clonic seizure managed by the paramedical team in charge of the transfer.

In the initial psychiatric examination, the patient was conscious, neglecting his body care, and expressing persecutory remarks with an interpretative mechanism. Those thoughts were experienced with a low affective charge. The patient also presented a disorganization syndrome made of impenetrability of the motives of his bizarre behaviors and of an ambivalence.

The neurological examination, the biological assessment, and the electroencephalogram (EEG) were without any particularity.

**Figure 1:**
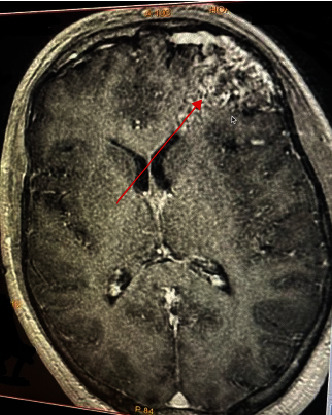
T1 fat-saturation after gadolinium injection: axial slice showing the AVM on the left frontal lobe as shown with the red arrow.

**Figure 2:**
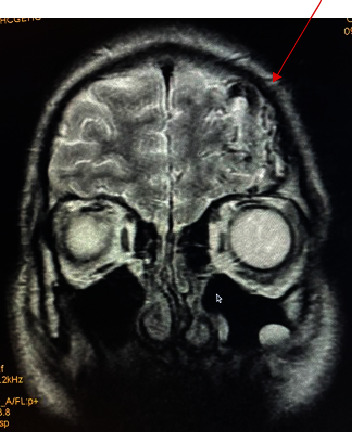
T2 coronal slice showing the 5 × 4 cAVM (red arrow).

However, the cerebral CT scan showed “a serpiginous structure in the left frontal subarachnoid spaces, infiltrating into the bottom of the sulcus, spontaneously hyperdense, and containing a dotlike calcification. Further imaging by cerebral angiography revealed a lesion on the left frontal side on the axe, formed by serpiginous structures in T1 and T2 asignal, taking contrast, corresponding to vascular structures draining to the superior longitudinal sinus, measuring 5 × 4 cm respecting the midline” (Figures 1 and 2).

The neurosurgical team decided to withhold treatment because of the size of the grade 2 abnormality. Indeed, an intervention presented more risk than well-being.

He was put on sodium valproate, then was objectified for a complete clearing of the psychiatric symptoms without the use of antipsychotics.

The patient was discharged with a follow-up neurosurgery in ambulatory.

One year later, he was still scheduled for surgery, clinically the patient did not present dissociative syndrome, nor seizures, he is still followed in ambulatory.

**Figure 3:**
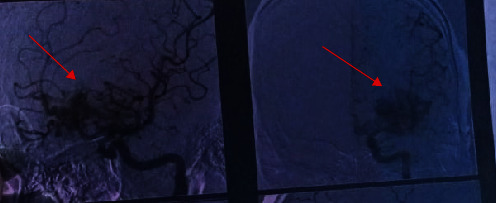
Brain arteriography showing the nidus of the cAVM.

Radiologically, the follow-up CT angiography and cerebral arteriography did not reveal any aggravation but it still shows a high-flow left anterior basifrontal cAVM (Figure 3).

## 3. Discussion

### 3.1. The Patient

The patient was immediately referred to the psychiatric emergency room based on the existence of a common psychiatric profile; he was a young person, with no prior history of a medical illness or delirium. This approach was only reconsidered after the detection of neurological signs indicating the possibility of the brain's involvement. And thus, motivating the physicians to resort to clinical and radiological neurological examinations, leading to the discovery of a left frontal arteriovenous malformation.

**Figure 4:**
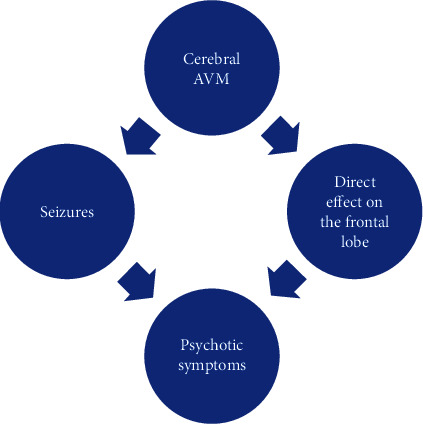
The two theories explaining the case.

Two hypotheses can therefore explain this clinical presentation: Would it be the vascular malformation that has directly caused the psychotic symptoms by its direct contact with the frontal lobe. Or is it through convulsions (Figure 4).

The treatment with sodium valproate leading to the clearing of the psychiatric symptoms somehow led us into believing the post-ictal origin of the symptoms, in despite of a normal EEG (which is quite frequent in epilepsies), this reflection was made since no other seizure was reported, and no other secondary psychotic symptoms were observed.

### 3.2. Cerebral Arteriovenous Malformations (cAVM)

An arteriovenous malformation (AVM) is a vascular illness characterized by a malformed vascular network inducing abnormal connections between cerebral arteries and veins. These abnormal connections induce an abnormal elevation of blood pressure in the malformation and in the veins draining the cAVM. The wall of these vessels is more fragile and therefore at risk of breach.

They are relatively rare with a prevalence of 0.06%–0.5% [6–10], while the prevalence of symptomatic cAVMs is at 0.01% [11, 12].

Cerebral AVMs can be complicated by cerebral hemorrhages (40%–50%) (lobar hematoma, subarachnoid hemorrhage, and ventricular hemorrhage) and seizures (20%–35%) most commonly generalized seizures (30% of all seizures) [13]. More rarely, blood deflection and locoregional hemodynamic changes secondary to the AVM can lead to ischemic brain injury [14].

The Spetzler–Martin grading system classifies AVMs into five grades according to its size, the venous drainage, and the eloquence. Grades 4 and 5 are called “high grade” and represent only 10% of all AVMs [15].

### 3.3. Postictal Psychosis (PIP)

It corresponds to the sudden appearance of acute psychotic symptoms of short duration after a burst of epileptic seizures, only 2% of people with epilepsy express a PIP.

They are classified with the ictal and peri-ictal psychoses when they are brief, and with the episodic interictal psychoses when they are extended.

Chronologically, the time gap between the seizure and the psychotic episode must be more than 24 hr and less than 7 days. Some authors report gaps of a few hours [16].

Concerning the duration of the psychosis, most authors report a duration of 3–14 days [17]. Extreme cases of 3 months have been reported [18]. This criterion is important especially in prospective studies, to differentiate postictal psychosis from interictal psychosis.

### 3.4. Psychiatric Symptoms and Brain's Abnormalities

Although the anatomical specificities of brain lesions giving place to neuropsychiatric symptoms have been well-studied, some uncommon presentations are still underdiagnosed. This is particularly the case for psychotic symptoms. It is not common for brain tumors, regardless of location, to present only with psychiatric symptoms [19]. Of the psychiatric symptoms indicative of brain injury, mood disorders are the most common (36%), while psychotic symptoms are found in 22% of cases [19]. In addition, previous studies have shown that depression is mainly associated with frontal lobe tumors, especially if they are located in the left frontal lobe [19–21]. However, rare cases of psychosis associated with left frontal lobe tumors have been reported, and our case is a good example [22, 23]. These symptoms are more classically observed in temporal lobe or pituitary tumors [21].

Indeed, 88% of masses causing psychiatric symptoms are located in the frontal region [24]. The frontal cerebral lobes are often a “silent” area, as benign tumors such as meningiomas or vascular malformations that outwardly compress these lobes may only produce changes in personality and cognitive functions. Signs that can go unnoticed.

On the psychiatric level, there are several organic pathologies that can take the mask of psychotic symptoms (delusions, hallucinations). The differential diagnosis is made by investigations guided by the findings of the initial clinical assessment. If infectious and toxic causes are the most common organic diagnoses suggested in the presence of a psychiatric picture, processes occupying the space in the frontal regions should also be considered.

Psychiatrists are then often the first to follow these patients, and the correct diagnosis may not be made until the tumor has grown to a considerable size and has begun to move adjacent brain structures [20].

### 3.5. Statistics of Our Patient

As we mentioned before, the frequency of symptomatic cAVMs is rare, estimated at 0.01% in the general population. In these rare cases, there is a 22% chance of showing psychotic symptoms.

Whereas if the symptomatic cAVM (0.01%) has expressed its influence through convulsions (second theory), which are only present in 30% of them, the risk of postictal psychosis is even less frequent (2%).

**Figure 5:**
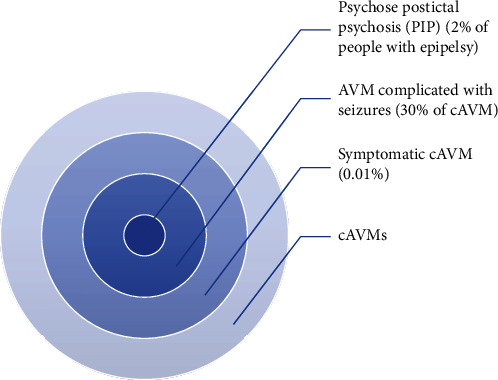
Rarity of the case (size not to scale).

All these numbers only translate the rarity of this clinical case (Figure 5).

## 4. Conclusion

Cerebral vascular malformations are a heterogeneous group of disorders with very different natural histories and clinical implications.

Once a patient has been diagnosed with a psychiatric disorder, his or her status is rarely reconsidered in search of possible organic causes.

Both psychiatrists and physicians should keep in mind that the response of psychiatric symptoms to psychotropic treatments does not exclude an underlying organic cause, and that if there is any doubt, the initial etiological workup should be thorough.

## Data Availability

Data sharing is not applicable to this article as no data sets were generated or analyzed during the current study.

## Abbreviations

cAVMs: Cerebral arteriovenous malformations
PIP: Postictal psychosis
CT angiography: Computed tomographic angiography.
